# Enhanced Hydrogel Materials: Incorporating Vitamin C and Plant Extracts for Biomedical Applications

**DOI:** 10.3390/molecules29112633

**Published:** 2024-06-03

**Authors:** Magdalena Kędzierska, Katarzyna Sala, Magdalena Bańkosz, Klaudyna Grzela, Piotr Potemski, Krzysztof Miernik, Bożena Tyliszczak

**Affiliations:** 1 Department of Chemotherapy, Copernicus Memorial Hospital of Lodz, Medical University of Lodz, 90-549 Lodz, Poland; magdalena.kedzierska@umed.lodz.pl (M.K.); piotr.potemski@umed.lodz.pl (P.P.); 2 Department of Materials Engineering, Faculty of Materials Engineering and Physics, Cracow University of Technology, 37 Jana Pawła II Av., 31-864 Krakow, Poland; klaudyna.grzela@kit.lukasiewicz.gov.pl (K.G.); krzysztof.miernik@pk.edu.pl (K.M.); bozena.tyliszczak@pk.edu.pl (B.T.); 3 Lukasiewicz Research Network—Cracow Institute of Technology, Zakopiańska 73 St., 30-418 Krakow, Poland

**Keywords:** hydrogel materials, vitamin C, antioxidant properties, sorption capacity

## Abstract

In recent years, the utilization of natural components has become crucial across various industries, including medicine. Particularly in biomedical contexts, hydrogel materials are of significant importance. Therefore, the objective of this research was to develop and analyze hydrogel materials infused with vitamin C. A key focus of this study was to conduct multiple syntheses with varying levels of vitamin C to explore the feasibility of creating materials with adjustable properties. The produced hydrogels underwent comprehensive physicochemical evaluation. The findings of this examination verified the correlation between the vitamin C content and the specific characteristics of the hydrogels. It was determined from these results that the samples displayed both sorptive and antioxidative capabilities, enabling their potential application in wound dressings or other biomedical uses. A notable benefit of these hydrogels is their adaptability, allowing for modifications to achieve desired attributes tailored to particular applications.

## 1. Introduction

Modern medicine is constantly striving to improve the application and therapeutic effectiveness of drugs, while minimizing their potential side effects and unwanted interactions. In the context of this pursuit, hydrogels represent an innovative tool that can be used as a drug carrier and delivery system for active substances into the body [1]. Hydrogels are characterized by their ability to absorb and retain water within the polymer network, making them ideal for biomedical applications such as dressings and drug release [2,3]. The process of creating hydrogel materials can be based on a variety of ingredients, including natural polymers such as chitosan. Chitosan, a derivative of the natural polysaccharide chitin, is characterized by its biocompatibility and non-toxicity, making it an attractive material for biomedical applications, including hydrogel production [4]. One of the key features of chitosan is its ability to form hydrogels by reacting with suitable cross-linking agents, such as aldehydes, which lead to the formation of a three-dimensional polymer network. This polymer network is characterized by a high capacity for water absorption and retention, making chitosan-based hydrogels excellent drug carriers and active agent release systems. In addition, chitosan exhibits the ability to interact with various biologically active compounds, which can lead to improved hydrogel properties [5,6]. For example, chitosan can form complexes with additional therapeutic agents, such as vitamin C or plant extracts, which can lead to improved stability, bioavailability, and therapeutic efficacy. In addition, chitosan also exhibits biological activities, such as antimicrobial and anti-inflammatory activity, making it an attractive ingredient for hydrogels intended for applications in the treatment of skin infections and wound healing. It is also worth noting that chitosan has the ability to induce regenerative processes in tissues, which can contribute to faster wound healing and improved tissue regeneration processes, highlighting its importance in the production of hydrogels for biomedical applications [7,8,9]. The possibility of modifying hydrogels through the addition of active ingredients, such as vitamin C and plant extracts, opens up new perspectives in the field of topical therapy and controlled drug release [10]. Vitamin C, known mainly for its antioxidant properties, can be effectively used to modify hydrogels, which could lead to the development of innovative solutions in medicine. Its local administration via a hydrogel can enable concentrated delivery of the substance to the site of action, which can be particularly beneficial in the treatment of various diseases and pathological conditions [11,12]. Selected plant extracts, such as Calendula officinalis and Arnica montana, are known for their anti-inflammatory and wound-healing properties [13,14]. Their modification of hydrogels can increase their effectiveness and speed, which can significantly improve the healing process and reduce the risk of infection [15]. Arnica montana and Calendula officinalis extracts are natural sources of compounds with proven anti-inflammatory, antibacterial, and healing properties [16,17,18,19]. Their incorporation into hydrogels can lead to the creation of a therapeutic platform that not only relieves symptoms of inflammation and speeds up the wound healing process, but also minimizes the risk of infection. The addition of vitamin C (ascorbic acid) to hydrogels modified with arnica montana and calendula extracts is an important innovation. Vitamin C, known for its strong antioxidant properties, can support the skin’s regenerative processes by neutralizing the effects of free radicals and stimulating collagen synthesis. Combined with the antibacterial and anti-inflammatory properties of plant extracts, vitamin C can create a synergistic effect, enhancing the therapeutic effect of hydrogels.

In this article, we will focus on the study of hydrogels modified with vitamin C and selected plant extracts, analyzing their therapeutic potential and applicability in clinical practice. By understanding the mechanisms of action of these modifications, we will be able to better assess their value and potential in the context of modern medicine, thus opening new perspectives in topical therapy and delivery of active substances to the body. In order to evaluate the applicability of hydrogel materials enriched with natural components, selected compositions were synthesized and their physicochemical analysis was carried out by determining the degree of swelling and roughness profile. In addition, studies of antioxidant properties and incubation in fluids simulating conditions in the human body were carried out. In turn, Fourier transform infrared spectroscopy was used to analyze the chemical structure of the polymer systems obtained.

## 2. Results and Discussion

### 2.1. Analysis of Sorption Capacity

**Figure 1:**
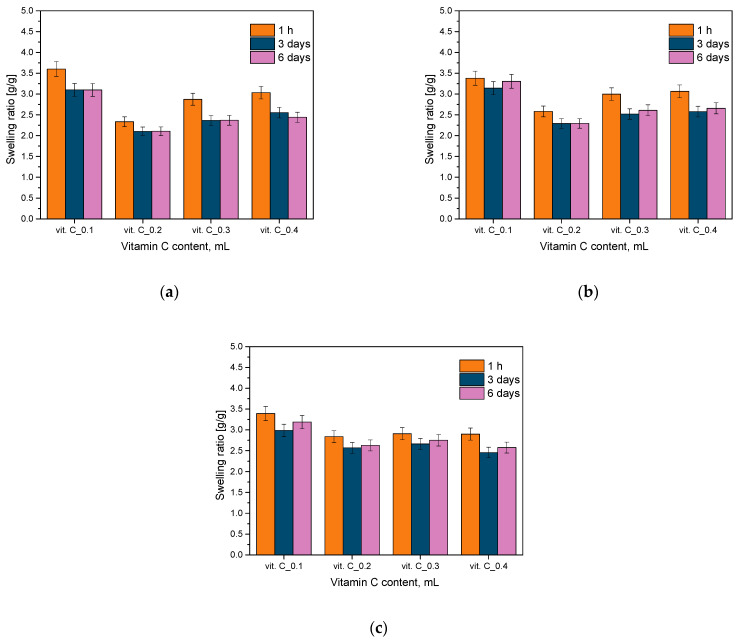
Outcomes of the absorption capacity study in distilled water (**a**), SBF liquid (**b**), and Ringer’s solution (**c**) (*n*—number of experiments, *n* = 3).

To investigate and assess the sorption capabilities of hydrogel materials, a swelling test was performed. This research considered the impact of varying concentrations of vitamin C. The outcomes from this analysis, conducted in simulated body fluid (SBF), Ringer’s solution, and distilled water, are illustrated in the graphs provided below (Figure 1).

Investigating the sorptive capacity of hydrogel materials in the context of biomedical applications is important for assessing their functionality and therapeutic efficacy. The ability of hydrogels to absorb water and biologically active substances is crucial for controlling drug release, regulating moisture in the surrounding environment, and evaluating their capacity to absorb body fluids. In addition, the absorption capacity can affect the flexibility and adaptation of the hydrogel to the shape of wounds, as well as its stability and durability under different environmental conditions. As a result, the study of sorption enables a better understanding and optimization of hydrogel materials, resulting in improvements in their therapeutic efficacy and patient comfort in biomedical applications. All the tested hydrogel materials showed a swelling coefficient. The swelling kinetics showed the highest jump in sorption capacity after 1 h of incubation, followed by a gradual stabilization of the value with time. The initial jump in sorption capacity could be related to the interaction of the polymer matrix with the liquid. At a later time, most samples showed a decrease in the swelling coefficient, which may be related to saturation processes and the release of active ingredients from the hydrogel matrix. The analysis indicated that the highest values of the swelling coefficient were observed for the Ringer’s solution. This may be related to its chemical composition. Ringer’s solution is an aqueous solution of mineral salts, which has similar properties to the body fluids of the human body. In addition, due to the presence of salts and ions in its composition, this can influence the absorption processes of hydrogel materials.

In this study, where the samples contained the least amount of vitamin C and plant additives, the highest value of the swelling coefficient was observed compared to samples with more of these modifiers. There are several possible reasons for this phenomenon, which may be due to the effect of the additives on the properties of the hydrogel material. First, the introduction of plant additives and vitamin C may lead to chemical interactions between hydrogel components and additives. These interactions can affect the structure of the polymer network and the formation of additional water-binding sites, which alter the material’s ability to absorb liquids. Next, plant additives and vitamin C can modify the structure and morphology of the hydrogel material, which in turn can increase its specific surface area and water absorption capacity. These structural changes can result from both physical and chemical interactions between the additives and the hydrogel material. In addition, the chemical properties of the additives, such as increased hydrophilicity or ability to form complexes with water, can promote the sorption process in the hydrogel material. This can lead to an increase in its ability to absorption water, as reflected by high swelling index values. In the case of samples containing higher amounts of vitamin C and plant additives, there is a possibility that their concentrated doses may have reduced the swelling process. This could be due, for example, to competitive water binding or reduced availability of swelling sites in the hydrogel structure. Both the amount and type of additives modifying the hydrogel material can significantly affect its sorption capacity, which is an important factor in assessing its suitability for biomedical applications.

### 2.2. Results of the Incubation Study

**Figure 2:**
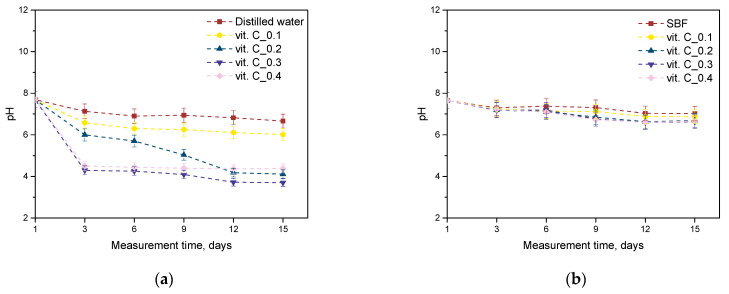

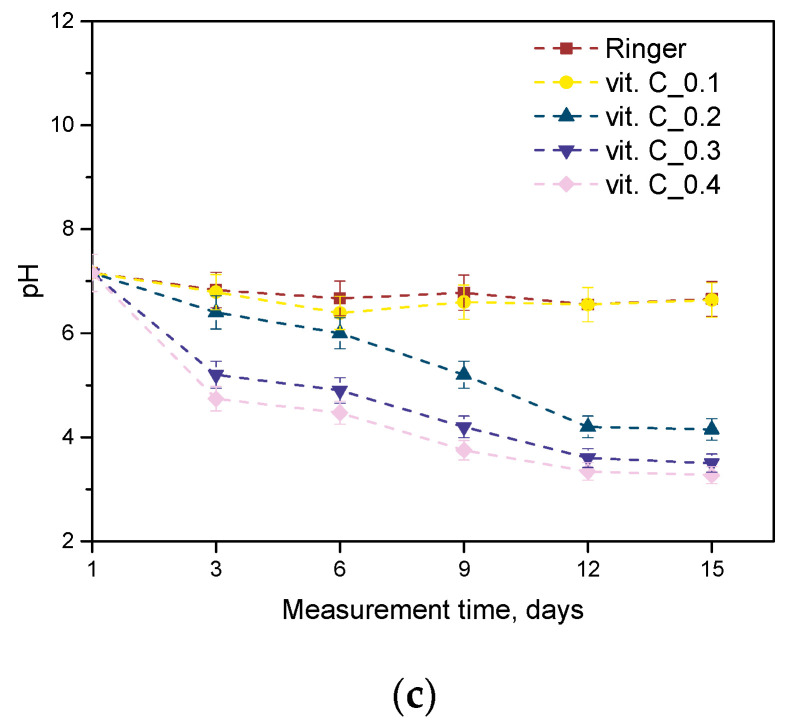
Outcomes of incubation tests in distilled water (**a**), SBF liquid (**b**), and Ringer’s solution (**c**) (*n*—number of tests, *n* = 3).

Tests were conducted to measure the hydrogen ion activity (pH) in the fluid where the hydrogel matrices were incubated over a 15-day period. The findings are presented in the graphs below (Figure 2).

Performing incubation analysis of hydrogels placed in simulated body fluids to determine the change in pH of the incubation environment is crucial to assess the stability and reactivity of the material under physiological conditions. The change in pH of the environment can result from the interaction of the hydrogel with chemicals in the body fluids, as well as from degradation processes or chemical reactions taking place in the material’s structure. Incubation analysis also makes it possible to monitor the stability of the material over time and identify any degradation processes or chemical reactions occurring in the hydrogel structure. Understanding how changes in the pH of the incubation environment affect the durability and functionality of the material is important to ensure its safe and effective use in biomedical applications. As the amount of additives increases, such as vitamin C and plant extracts, the pH of the Incubation environment has been shown to gradually decrease. This Is due to the Introduction of organic acids contained in these additives into the environment, leading to the accumulation of hydrogen Ions and lowering the pH. Vitamin C, also known as ascorbic acid, can be seen In such a context as an acid-forming agent, which in higher concentrations can lead to a decrease in pH. Analyzing the pH change in the samples provides an understanding of the effects of different additive concentrations on the acid–base balance of the incubation environment and on the structural stability of the hydrogel material. It is worth noting that changes in pH can affect various physicochemical processes in the material, such as the breakdown of the polymer network or changes in the degree of hydration, which can have important implications for its sorption properties, stability, and therapeutic efficacy. The analysis clearly confirms the possibility of releasing active substances from inside the polymer network resulting in lower pH values. The study proves that the higher the amount of plant additives and vitamin C, the progressively lower the pH values of the incubation environment. However, the exact pH changes may depend on a number of factors, such as the type of additives used, their concentration, and the specifics of the hydrogel material.

### 2.3. Results of Antioxidant Activity Analysis

**Figure 3:**
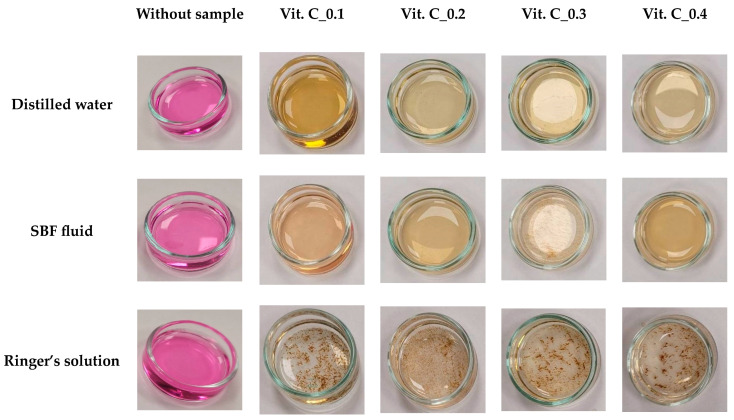
Results of antioxidation measurement of samples of distilled water, SBF solution, and Ringer’s fluid containing increasing amounts of vitamin C.

To confirm the results from the incubation study regarding the potential release of active compounds, an analysis of antioxidant activity was conducted. The incubation fluids, which previously contained hydrogel materials, were examined. Additionally, an incubation fluid without any hydrogel material was used as a control sample. The results of this analysis are displayed in Figure 3.

The antioxidant potential was evaluated in samples both containing and lacking vitamin C. During the experiments, the duration required for the KMnO_4_ solution to fade to a brownish-yellow shade, signaling the reduction of MnVII+ ions to MnII+, was recorded. This change was observed to occur within seconds for all tested materials. A control test was also conducted using the incubation fluid alone, where no sample was placed, and no color change was noted in this case. Hence, it can be inferred that the incubation outcomes align with the antioxidant property analysis. Active substances that demonstrate antioxidant capabilities are being released from the hydrogel materials within just a few seconds. Antioxidants are defined as compounds that, even in minimal amounts, safeguard the body from free radical damage. Furthermore, antioxidants impede oxidation reactions, support the body’s defenses against microbial threats, and prevent the multiplication of free radicals—electron-deficient molecules that extract electrons from healthy cells in search of stability. Given the critical role of substances like vitamin C as antioxidants, the study also examined the interaction of released compounds from the hydrogel materials with potassium permanganate (VII). The analyses, performed with varying concentrations of additives across different biological fluids, revealed significant relationships between the sample compositions and their antioxidant capacities. The most important finding of the study was that the hydrogel-containing samples, which showed antioxidant activity, tended to discolor in the fluids tested simulating the environment of the human body. The discoloration process was more pronounced in samples where the content of additives systematically increased. However, samples without the addition of hydrogel did not show discoloration, suggesting that distilled water or Ringer’s fluid alone do not exhibit antioxidant properties, and that the results are due to the release of active substances by the hydrogel material. Analysis of the color of the samples in different liquids showed that the prevailing chemical environment had a significant effect on the processes in the samples. In an acidic environment such as Ringer’s liquid, potassium permanganate (VII) was reduced to manganate (II), resulting in a precipitate in the samples. In contrast, brown-colored samples showed reduction to manganese (IV) oxide, suggesting that the environment was neutral or slightly alkaline.

### 2.4. Results of Infrared Spectroscopy Analysis

**Figure 4:**
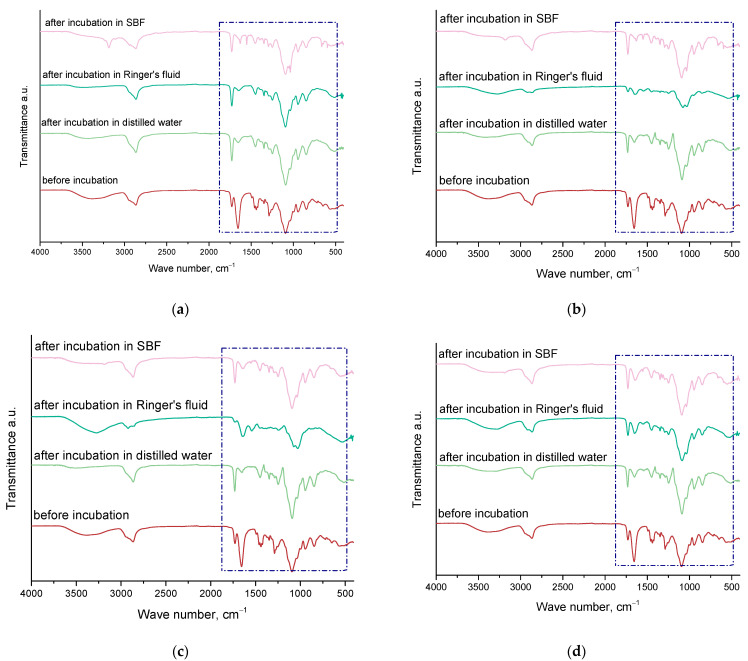
FT-IR spectroscopic analysis results for samples containing 0.1 (**a**); 0.2 (**b**); 0.3 (**c**); 0.4 (**d**) vitamin C.

Spectroscopic examination was performed on the materials both right after their synthesis and after the incubation period. The purpose of this analysis was to evaluate how the incubation process affected the structural integrity of these materials. The results of this study are depicted in Figure 4.

Table 1 displays the detected absorption bands, along with the associated types of vibrations and characteristic bonds.

Infrared spectroscopy (FT-IR) analysis performed on the hydrogel materials enabled the identification of characteristic absorption bands associated with individual components and revealed changes in the structure of the materials during incubation. The absorption band associated with stretching vibrations of -OH groups in the range of 3182–3503 cm^−1^, characteristic of almost all material-building components, i.e., PVP, chitosan, and all additives, was determined. The presence of -CH bonding in the 2863–2942 cm^−1^ range, derived from chitosan and arnica montana, was confirmed. The band in the range of 1724–1741 cm^−1^, representing the C=O bond, was characteristic of calendula officinalis and PEGDA 700. The C=C bond, present in the range of 1629–1655 cm^−1^, was identified as originating from vitamin C and PEGDA 700. Also detected was a deformation bond -CH in the range 1421–1455 cm^−1^, which was present in both vitamin C and PEGDA 700. The last bond identified was C-O in the range 1035–1096 cm^−1^, characteristic of marigold. FT-IR analysis showed that the materials changed in structure during incubation. The absorption bands associated with the C=C and C-H deformation bonds disappeared slightly, suggesting the release of active components from the polymer network. In addition, the -OH band changed, with it disappearing in some cases and becoming more intense in others, especially in the SBF fluid. The disappearance of this band may be due to the release of additives from the material structure. Moreover, no significant changes were observed that could affect the degradation of the hydrogel material during incubation analysis.

### 2.5. Optical Microscope Observations

**Figure 5:**
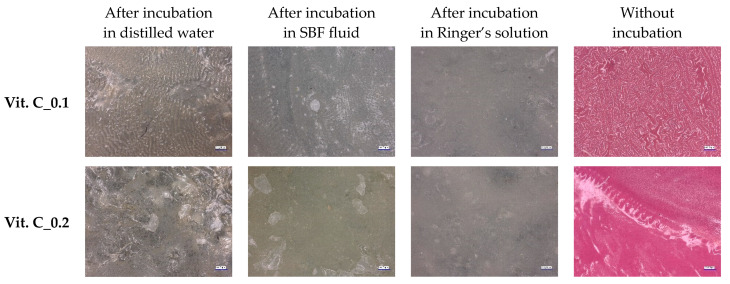

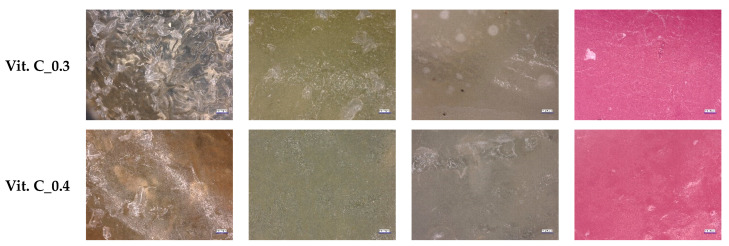
Results of microscopic observations of samples before and incubation in fluids simulating the environment of the human body (at magnification ×100).

A microscopic examination using an optical microscope was conducted to visualize and compare the surfaces of the hydrogel materials. The images captured during these observations are presented in Figure 5 at magnification ×100.

Microscopic analysis of the tested vitamin-C-containing hydrogel materials made it possible to observe significant changes in structure and showed the effect of incubation on their morphology and appearance. Based on the above microscopic images, it was found that non-incubated samples were characterized by a pink color, while after incubation they became colorless. After the samples were incubated, their color was white or colorless; however, they took on a pink hue during the drying process. It is possible that the color change during drying was due to the sensitivity of vitamin C to oxygen and UV radiation in aqueous solutions. The colorless color of the dry samples after incubation suggests the release of active substances during soaking, which is evidence of the release of substances from their structure. Analysis of the surface of the samples in SBF and Ringer’s liquids showed their smoothing compared to the dry samples. More undulations can be observed on the surface of samples incubated in distilled water. During swelling, the polymer network was loosened and additives were leached out, which may have resulted in a smoother surface in the SBF and Ringer’s liquids. However, no drastic changes in the structure of the material or its destruction were observed, indicating its stability.

### 2.6. Digital Microscope Observations and Roughness Profile Analysis

**Figure 6:**
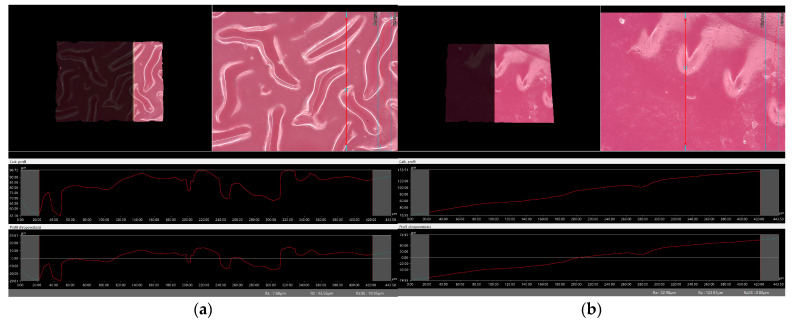

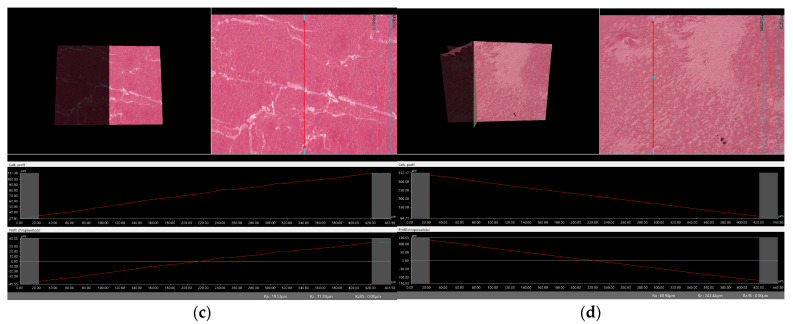
Roughness profile image for unincubated sample obtained: (**a**) vit.C_0.1, (**b**) vit.C_0.2, (**c**) vit. C_0.3, (**d**) vit.C_0.4 (500× magnification).

**Figure 7:**
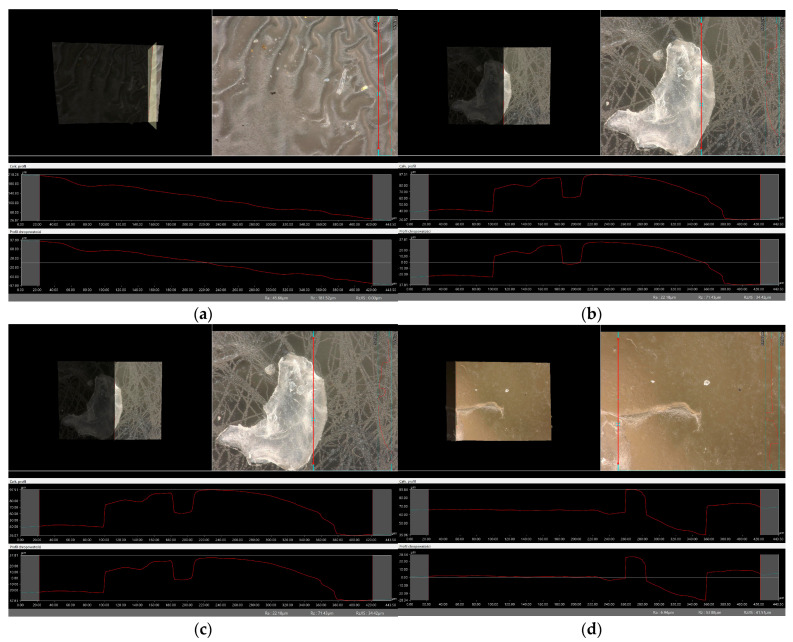
Roughness profile image for samples incubated in distilled water obtained: (**a**) vit.C_0.1, (**b**) vit.C_0.2, (**c**) vit. C_0.3, (**d**) vit.C_0.4 (500× magnification).

**Figure 8:**
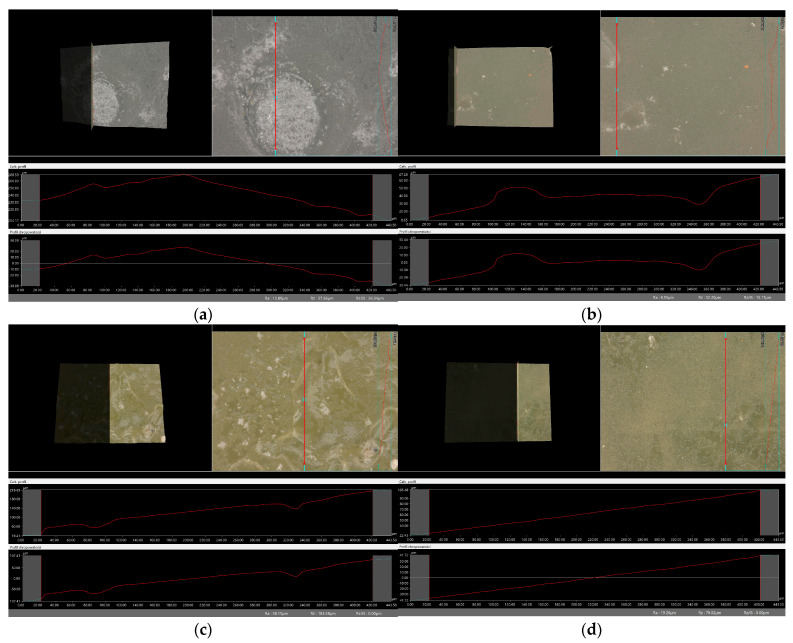
Roughness profile image for samples incubated in SBF liquid obtained: (**a**) vit.C_0.1, (**b**) vit.C_0.2, (**c**) vit. C_0.3, (**d**) vit.C_0.4 (500× magnification).

**Figure 9:**
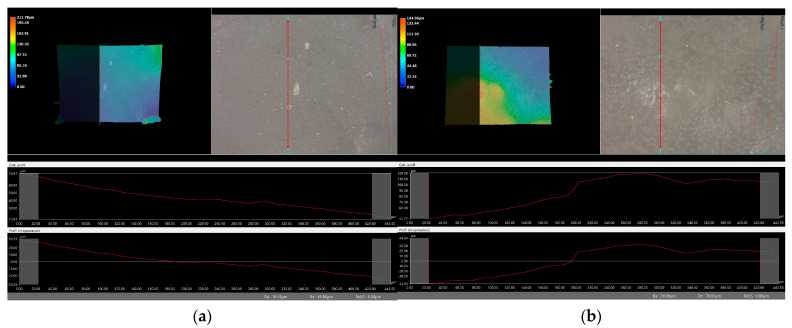

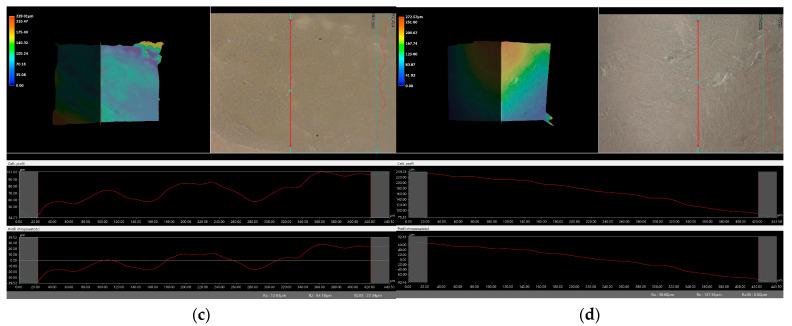
Roughness profile image for samples incubated in Ringer’s solution obtained: (**a**) vit.C_0.1, (**b**) vit.C_0.2, (**c**) vit. C_0.3, (**d**) vit.C_0.4 (500× magnification).

Subsequently, the microscopic observations were enhanced by employing a digital microscope, which facilitated the assessment of the surface roughness profiles of the hydrogel materials. The outcomes of this analysis are documented in Table 2 and Figure 6, Figure 7, Figure 8 and Figure 9.

Surface roughness measurements were conducted for all the materials produced. The values determining the fundamental roughness parameter, Ra (absolute average relative to the base length), are listed in Table 2.

The roughness profile determined by the Ra parameter was within the range of 6–60 µm for all samples both immediately after synthesis and incubation. There was no clear relationship between the roughness of incubated and dried samples immediately after synthesis. The lack of a clear relationship between the roughness profile of the hydrogel samples obtained after synthesis and the hydrogels after the incubation test may be due to various factors affecting the morphology and structure of the material, both during the synthesis process and during incubation. First, the incubation process can lead to structural and morphological changes in the hydrogel material. The effect of changing the pH of the incubation environment, the presence of chemicals in body fluids, or degradation processes can cause modifications in the hydrogel structure that will not necessarily be reflected in the roughness profile during the synthesis itself. In addition, differences in environmental conditions between synthesis and incubation can lead to different reactions and processes in the material. Factors such as temperature, humidity, or the presence of chemicals can be different during the two stages, which can affect differences in the structure and morphology of the material. It is noteworthy that the analysis showed no significant effects indicating the breakdown and degradation of the polymer matrix subjected to incubation analysis.

### 2.7. Determination of Antioxidant Properties by the DPPH Radical Method

This was followed by the DPPH radical method, which complemented the evaluation of the antioxidant capacity of the tested materials. These results are presented in Table 3.

**Figure 10:**
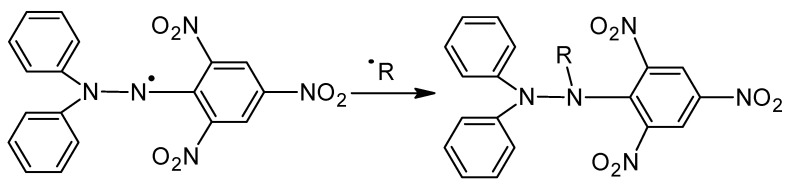
The DPPH radical reaction.

An analysis of the conducted studies on hydrogels containing different amounts of vitamin C in the context of their percentage inhibition by the DPPH radical method indicated a significant relationship between vitamin C content and antioxidant activity. It was observed that the higher the concentration of vitamin C in the hydrogel, the higher the percentage of inhibition, suggesting that vitamin C may have a crucial role in neutralizing free radicals. Vitamin C, being a strong antioxidant, can react with DPPH radicals, reducing their activity. As a result of this reaction, the color intensity of the DPPH solution is reduced, which is measurable by spectrophotometry. In the study, the more vitamin C that was present in the hydrogel, the more DPPH radicals were neutralized, which translated into a higher percentage of inhibition. The reaction of the DPPH radical is shown in Figure 10.

The confirmed relationship indicates that vitamin C content can have a significant impact on the antioxidant properties of hydrogels. Higher concentrations of vitamin C may lead to more effective neutralization of free radicals, which may be beneficial especially in the context of therapeutic applications, where protection against oxidative stress is crucial for tissue and cell health. However, potential limitations related to the solubility and stability of vitamin C in a particular hydrogel system, as well as possible interactions with other hydrogel components that may affect overall antioxidant activity, must also be considered. However, for all the materials tested, the percentage of inhibition was above 80% which indicates high antioxidant capacity and is in line with the results presented by other researchers [20,21].

### 2.8. Determination of the Release of Vitamin C

**Figure 11:**
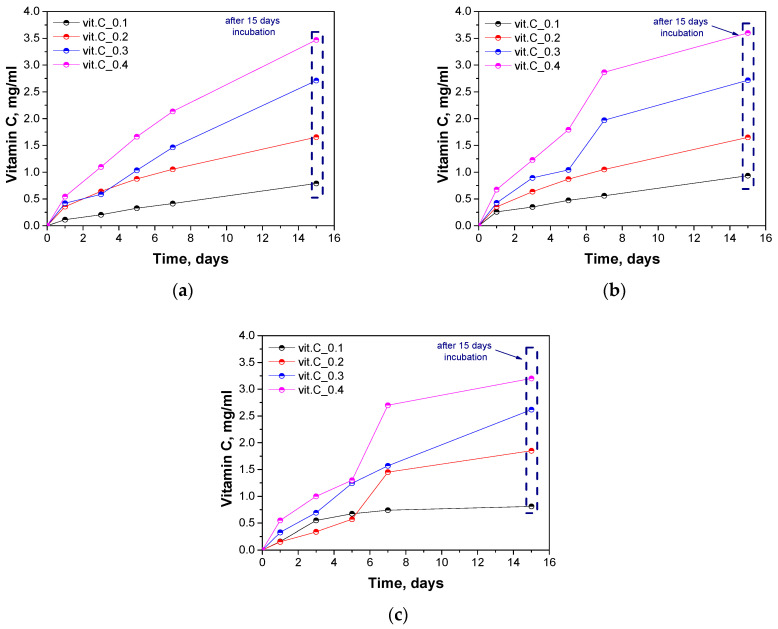
Results of vitamin C release from hydrogel samples in distilled water (**a**), SBF fluid (**b**) and Ringer solution (**c**).

Vitamin C release was determined using a spectrophotometric method. The results of release over time for samples placed in different incubation fluids are presented in Figure 11.

Based on the analysis, the ability to release the active substance from the hydrogel material was confirmed. The concentration of released vitamin C increased depending on the initial content. Samples containing the highest amount of vitamin C released the active substance at the highest rate and in a shorter time. With time, there was an increase in the concentration of the released active substance. This is a satisfactory result, as a hydrogel material with prolonged release kinetics was obtained. Vitamin C was not released rapidly on the first day of the test, which proves that the parameters of synthesis were well chosen. The results obtained represent the receipt of a promising hydrogel material capable of prolonged release of an antioxidant active substance.

## 3. Materials and Methods

### 3.1. Materials

Poly(vinylpyrrolidone) (powder, average mol wt. 58,000), chitosan (degree of deacetylation, 75–85%), diacrylate poly(ethylene glycol (crosslinker, average molecular weight Mn = 700 g/mol), and 2-hydroxy-2-methylpropiophenone (photoinitiator, d = 1.077 g/mL, 97%) were purchased from Sigma Aldrich (Darmstadt, Germany). Vitamin C in solid form was purchased from Stanlab (Lublin, Poland). In turn, Calendula officinalis and Arnica montana were purchased from Herbal Pharmaceuticals (Glogow Malopolska, Poland).

### 3.2. Synthesis of Hydrogel Materials

The synthesis process was initiated by diluting a 99.5% solution of acetic acid to a concentration of 0.05%. Solutions of chitosan and a 10% solution of PVP (polyvinylpyrrolidone) were prepared accordingly. These solutions were then combined in a specific sequence. Following this, a crosslinking agent, poly(ethylene glycol) diacrylate (PEGDA), was introduced to the mix, and then a photoinitiator, specifically 2-hydroxy-2-methylpropiophenone, was added. The mixture was then transferred to a Petri dish and exposed to UV light using an EMITA VP-60 lamp (Famed, Lodz, Poland) with a power of 180 W and a wavelength of 320 nm. The sample was irradiated for 120 s. Following the irradiation, the material was dried and prepared for subsequent analysis. A similar synthesis procedure was applied for samples that included herbal infusions and vitamin C, with these components being added prior to the crosslinking agent and photoinitiator. The composition of each hydrogel is detailed in Table 4. Vitamin C was added by weighing out the appropriate amount and dissolving it in a base solution consisting of polymers (chitosan and PVP). Plant extracts, on the other hand, were prepared by aqueous extraction of dried reagents (dried calendula flower and dried arnica basket). Extraction was carried out at 80 °C in water at a mass ratio of 5 g of dried substance per 50 mL of distilled water.

### 3.3. Sorption Capacity Analysis

The primary aim of the research was to ascertain the sorption capabilities of the hydrogel materials under investigation, particularly in terms of swelling. In the context of therapeutic dressings, it is paramount for the dressing material to possess sufficient swelling capacity, allowing it to absorb wound exudate effectively. To conduct the swelling analysis, small discs with a diameter of 1 cm were utilized. These discs underwent meticulous drying and weighing procedures. Subsequently, they were immersed in 50 mL of various liquids: Ringer’s solution, SBF liquid, and distilled water. Following a one-hour incubation period, the discs with excess liquid were drained, and they were reweighed. Comparable weight assessments were conducted after 3 and 6 days. To minimize measurement discrepancies, three repetitions were carried out for each sample. Finally, the swelling coefficient results from multiple trials were averaged to obtain a representative value for the sorption capacities of the hydrogel matrices. The degree of swelling was determined by calculating the sorption coefficient α, as outlined in Equation (1).

(1) $$\alpha =\frac{m_{t}-m_{0}}{m_{0}}\;$$

where

α—swelling ratio, g/g;
*m_t_*—mass of swollen sample after time “t”, g;
*m*_0_—mass of dry sample (before the study), g.

### 3.4. Incubation Studies in Simulated Body Fluids

The incubation studies aimed to investigate the interactions between the hydrogel matrix and solutions that simulate human physiological fluids. Changes in pH levels can reveal the leaching of uncrosslinked agents or the degradation of samples in these fluids, among other potential effects. For the experiments, hydrogel discs with a diameter of 1 cm were sterilized and placed in containers, each holding 50 mL of one of three selected fluids: Ringer’s solution, simulated body fluid (SBF), and distilled water. The samples were then incubated at 37 °C. pH measurements were taken using a CX-701 ELMETRON multifunctional device (Zabrze, Poland) over a 15-day period, with readings recorded every 3 days.

### 3.5. Analysis of Antioxidant Properties

To further enhance the incubation studies, an antioxidant capacity analysis was conducted. This analysis aimed to evaluate the potential antioxidant effects of substances released during the hydrogel materials’ incubation. All materials were included in this test, with an incubation fluid without any hydrogel serving as the control sample. The interaction between the test sample and potassium permanganate, a universal oxidizing agent, was selected for this analysis. Oxidation results in a color change in the samples. After a 15-day incubation period, the post-incubation fluids were separated from the hydrogel materials. A few drops of 0.1% potassium permanganate solution were added to 3 mL of each fluid, and the reaction was observed by measuring the time it took for the test sample’s color to change. If no color change occurred within one hour, the material was considered to lack antioxidant properties. Each test was performed in triplicate to ensure accuracy.

### 3.6. Characterization of the Chemical Structure of Hydrogel Materials via Fourier Transform Infrared (FT-IR) Spectroscopy

Spectroscopic analysis was conducted on all samples, including those that had not been incubated. The study examined a sample that was collected and dried immediately after synthesis, as well as those that had been incubated in their respective liquids for fifteen days. For this analysis, a Thermo Scientific Nicolet iS5 spectrometer (Waltham, MA, USA) with an ATR (attenuated total reflectance) attachment was used. Absorption spectra were recorded at room temperature, covering a wavelength range from 4000 to 600 cm^−1^.

### 3.7. Observation Using a Digital Microscope and Determination of Surface Roughness

Following the initial analyses, high-resolution images were captured using a Keyence 4K VKX-7000 digital microscope, based in Osaka, Japan. This device features a REMAX CEO (Danvers, MA, USA) and a 4K CMOS image sensor (OmniVision, Santa Clara, CA, USA), which offers high precision and magnification capabilities. The microscopic observation focused on the surface of hydrogel materials intended for biomedical use, providing a detailed examination of their structural and surface morphology. The photos were taken at ×100 and ×500 magnification. Roughness was determined for ×500 magnifications. This study makes it possible to comprehensively assess the quality and properties of hydrogel materials and identify any defects or inhomogeneous surfaces. This information is crucial for the further development and optimization of the materials, aiming to improve their effectiveness and safety in biomedical applications.

### 3.8. Determination of Antioxidant Properties by the DPPH Radical Method

To complement the evaluation of antioxidant properties, an analysis was carried out using the DPPH radical. The test was conducted for the filtrates obtained after a 15-day incubation analysis in simulated body fluids. A substance known as 2,2-diphenyl-1-picrylhydrazyl (DPPH) was used to evaluate antioxidant activity. First, a solution of DPPH was prepared at a concentration of 0.5 mM in methanol. It was then diluted to an absorbance level of about 0.9. The control sample was a previously prepared DPPH solution of 2 mL and the addition of 75 μL of methanol. Absorbance was measured with a V-5100 spectrophotometer at 517 nm (Genesys 180 UV-Vis spectrophotometer from Thermo Scientific, Loughborough, UK). The samples containing vitamin C were treated similarly, replacing 75 μL of methanol with the appropriate liquid obtained after incubation. The antioxidant capacity of the tested materials was determined by the following Equation (2):

(2) $$percentage\;of\;inhibition=\frac{A_{0}-A_{s}}{A_{0}}\;$$

where

*A*_0_—absorbance of DPPH solution;
*A_s_*—absorbance of sample.

### 3.9. Determination of the Release of Vitamin C

Determination of vitamin C release was performed according to the procedure proposed by Mushtaq et al. [22]. Briefly, a spectrophotometric method based on the principle of reducing iron III ions to iron II and forming a complex called ferroin using ascorbic acid in the presence of 1,10-phenanthroline was used. The absorbance of the resulting red Fe(II)-phenanthroline (Fer-roin) complex was measured at 430 nm. From the calibration curve performed, the amount of vitamin C released was determined. The release was carried out at specific time intervals. For this purpose, a sample of the hydrogel material was placed in the selected incubation fluid and the filtrate was collected at the specified time for the release test. Measurement after 15 days was made on the basis of previously obtained filtrate used to determine the antioxidant properties.

## 4. Conclusions

- Studies of sorption capacity indicated that differences in the amount and nature of additives used in hydrogel materials have a significant impact on their absorption capacity, which is an important factor in assessing their suitability for medical applications.
- On the basis of the analysis of antioxidant properties and incubation studies, the possibility of releasing active substances with antioxidant activity was proved. A reduced pH value indicates the release of vitamin C and plant extracts from the polymer matrix; in turn, the reaction with potassium permanganate confirmed the antioxidant properties of the released substances.
- During incubation, no significant changes affecting the degradation of the hydrogel material were observed. Therefore, FT-IR analysis demonstrated the dynamic nature of the hydrogel materials and their compatibility with the incubation environment, highlighting their potential for biomedical applications.
- There was no clear relationship between the roughness profile of hydrogel samples obtained after synthesis and hydrogels after incubation testing. This result may be due to various factors affecting the morphology and structure of the material, both during the synthesis and incubation processes. The incubation process can lead to structural and morphological changes in the material due to, among other things, changes in the pH of the incubation environment or the presence of chemicals. Differences in environmental conditions can lead to different reactions in the material. It is noteworthy, however, that the analysis showed no significant effects indicating the breakdown and degradation of the incubated polymer matrix.
- The findings from the study indicated that the samples demonstrated sorptive and antioxidant capabilities, making them suitable for further development as wound dressings. A significant advantage of these materials is their adaptability; they can be modified to achieve specific properties tailored to their intended applications.

## Figures and Tables

**Table 1:** Overview of characteristic bonds and vibrations associated with the observed absorption band values.

Wave Number [cm^−1^]	Vibration Type	Characteristic Binding
3182–3503	Tensile	O-H
2863–2942	Stretching symmetric and asymmetric	C-H
1724–1741	Tensile	C=O
1629–1655	Tensile	C=C
1421–1455	Deformation (bending)	C-H
1035–1096	Tensile	C-O

**Table 2:** Results of surface roughness analysis of hydrogel materials.

Sample Number	Ra, µm Unincubated Sample	Ra, µm SBF Liquid	Ra, µm Ringer’s Solution	Ra, µm Distilled Water
vit. C_0.1	7.68	13.89	10.13	45.66
vit. C_0.2	32.90	8.55	24.99	22.18
vit. C_0.3	19.53	38.15	12.63	32.78
vit. C_0.4	60.94	19.26	38.60	6.94

**Table 3:** Percentage of inhibition determined by the DPPH radical method.

Incubation Fluid	vit. C_0.1	vit. C_0.2	vit. C_0.3	vit. C_0.4
**Distilled water**	88.38 ± 0.16	91.69 ± 0.74	93.48 ± 0.86	97.03 ± 0.41
**SBF liquid**	84.14 ± 0.37	86.96 ± 0.80	8.39 ± 0.22	95.34 ± 0.28
**Ringer’s solution**	85.75 ± 0.49	87.04 ± 0.54	90.11 ± 0.78	98.45 ± 0.63

**Table 4:** Composition of hydrogel materials.

Base Solution *	PEGDA 700 g/mol, [mL]	Photoinitiator, [µL]	Vitamin C, [g]	Calendula Officinalis, [mL]	Arnica Montana, [mL]	Sample Name
10	2.0	50	0.1	1.5	1.5	vit.C_0.1
			0.2			vit.C_0.2
			0.3			vit.C_0.3
			0.4			vit.C_0.4

* Base solution: 6 mL 10% PVP and 4 mL 0.5% chitosan.

## Data Availability

The data that support the findings of this study are contained within the article.
